# Editorial: Mobile and wearable systems for health monitoring

**DOI:** 10.3389/fdgth.2023.1196103

**Published:** 2023-04-20

**Authors:** Mohamed Elgendi, Richard Ribon Fletcher, Derek Abbott, Dingchang Zheng, Panicos Kyriacou, Carlo Menon

**Affiliations:** ^1^ Biomedical and Mobile Health Technology Lab, ETH Zurich, Zurich, Switzerland; ^2^ Mobile Technology Group, Department of Mechanical Engineering, Massachusetts Institute of Technology (MIT), Cambridge, MA, United States; ^3^ School of Electrical and Mechanical Engineering, University of Adelaide, Adelaide, NSW, Australia; ^4^ Research Centre for Intelligent Healthcare, Coventry University, Coventry, UnitedKingdom; ^5^ School of Engineering, City University of London, London, United Kingdom

**Keywords:** mHealth, wearables, remote health monitoring, health data science, health analytics, digitalhealth, mobile computing, Artificial intelligence in healthcare

**Editorial on the Research Topic** Mobile and wearable systems for health monitoring

Over the past decade, there has been a growing interest in using mobile and wearable systems for healthcare applications. To contribute to this field, our Research Topic on *Mobile and Wearable Systems for Health Monitoring* has collected a variety of contributions, ranging from original research to reviews and perspectives, all focused on exploring new or existing methods, protocols, and models for health monitoring. These contributions cover various topics, including machine learning algorithms, data quality, and using smartphones and wearable devices to assess COVID and mental health. Additionally, our initiative encouraged multidisciplinary approaches to explore innovative health applications, resulting in exciting studies that examine new wearable and mobile devices in a hospital setting. Our Research Topic aims to provide insights into the potential of mobile and wearable systems for healthcare applications and inspire further research in this field.

The COVID-19 pandemic emphasized the importance of leveraging digital infrastructure for remote patient monitoring, which could be facilitated through the use of wearable sensors. The review article by Seshadri et al. showed that by using predictive platforms, these devices could potentially aid in disease detection and monitoring on an individual and population level. Public health officials and researchers could also use anonymous data to track and mitigate the spread of the virus. Their manuscript highlights the potential of clinically relevant physiological metrics from commercial devices in monitoring the health, stability, and recovery of COVID-19+ individuals and front-line workers. This paper helps encourage front-line workers and engineers to develop digital health platforms for monitoring and managing the pandemic.

It is challenging to evaluate the reliability of a wearable device in healthcare. The work by Santos et al. discusses how a wearable ambulatory monitoring system was optimized to monitor COVID-19 patients in isolation wards in the United Kingdom. The system used a chest patch and pulse oximeter to estimate and transmit continuous vital sign data from patients to remote nurse bays, minimizing the risk of infection for nursing staff. The system operated through a secure web-based architecture and fault-tolerant software strategies, allowing for remote monitoring of patients. The plan was used for almost half of the patients in the isolation ward during the peak of hospital admissions in the local area and was found to be effective in monitoring patients. An updated version of the system has also been used in subsequent waves of the pandemic in the UK. The implementation of wearable ambulatory monitoring systems represents a crucial step towards a safer and more effective way to monitor COVID-19 patients in isolation wards, significantly minimizing the risk of infection for nursing staff and opening a promising path for the future of healthcare.

As the use of wearable sensors grows, researchers have developed a range of wearable devices that collect data on our daily activities, such as movement, sleep duration, heart rate, skin temperature, and more, to monitor and analyze our mental health. The data collected by these devices are translated into patterns that can indicate symptoms of mental health disorders, such as depression, anxiety, and stress. Machine learning helps identify behavioral markers and the relationship between the raw sensor data and mental health conditions. The review by Sheikh et al. discusses the available smartphone-based, wearable, and environmental sensors that can be used to detect mental health conditions and provide a helpful tool for managing and treating them.

Cardiovascular disease, which is the leading cause of mortality worldwide, remains a popular field of research for new wearable sensor algorithms. Electrocardiogram (ECG) signals continue to be used throughout medicine and researchers have been enhancing the accuracy of automated heart disease diagnosis by exploring mathematical feature transformations for ECG signal segments. Liang et al.'s team tested six different mathematical transformation methods using 10-second ECG segments and found that applying the reciprocal transformation resulted in consistently better classification performance for normal and abnormal heartbeats. The cubic transformation was the second-best in terms of heartbeat detection accuracy. Surprisingly, the commonly used logarithmic transformation did not perform as well as the reciprocal or cubic transformations. By using the optimal transformation method, the reciprocal transformation, doctors can improve the accuracy of detecting normal and abnormal heartbeats by 35.6%. The researchers concluded that adding a simple data transformation step, such as the reciprocal or cubic, to the extracted features can significantly enhance current automated heartbeat classification, leading to better and faster diagnoses of heart diseases.

In addition to advances in algorithms, new data collection methods have also begun to emerge for cardiovascular disease, such as the regular monitoring of blood pressure for detecting hypertension and reducing the risk of cardiovascular disease. Since the traditional cuff-based method can be inconvenient and discourage regular monitoring. Smartphone sensors, such as video cameras, can detect arterial pulsations and be used to assess cardiovascular health. Researchers have developed advanced image processing and machine learning techniques to predict blood pressure using smartphones or video cameras. The review by Steinman et al. discusses the challenges associated with using smartphones in homes and clinics, but further testing is necessary under different conditions. This research shows that smartphones and video cameras have the potential to measure multiple cardiovascular metrics beyond blood pressure, which could significantly reduce the risk of cardiovascular disease.

The traditional functions of wearable sensors to measure physical activity and sleep (actigraphy) has now been extended to mobile and portable platforms that can be used in the home environment. Despite the recognized importance of sleep and circadian rhythm, conventional polysomnography (PSG) monitoring requires specialized equipment, making it unrealistic to assess the sleep stage in the home environment. The use of camera-based methods for sleep monitoring has increased in recent years; however, the published studies have mainly focused on adults. Kamon et al. developed an infrared camera-based monitoring system for children between 0‒6 years old. Extremely randomized decision trees (“Extra Trees”), an ensemble machine learning algorithm, were used to estimate the sleep stages from various information extracted from body movements. Comparable performance was achieved compared to simple PSG scoring, suggesting that their system could be potentially used as a non-contact sleep monitoring system for children at home.

Photoplethysmography (PPG) continues to be popular technique widely used in Digital and Wearable Health monitoring. However, there has yet to be a published consensus on signal quality expectations, especially for morphological PPG pulse analysis. Huthart et al. conducted a signal quality expectation survey with fellow international researchers in skin contact PPG measurements. They determined a consensus regarding the minimum recording length, the minimum number of undistorted pulses required, and the threshold proportion of noisy beats needed for the recording rejection. Their study provided initial recommendations to support the need to move toward improved standardization in measurement protocol and morphological pulse wave analysis and the need to gather repeatable and meaningful PPG data for implementing PPG sensing technology in Digital and Wearable Health devices.

In critical care settings, patients require continuous monitoring, but current methods do not capture important functional and behavioral indices. Advances in non-invasive sensing technology and deep learning techniques can transform patient monitoring by enabling continuous and granular monitoring of critical care measures. The paper by Davoudi et al. highlights current approaches to pervasive sensing in acute care, identifies limitations and opportunities, and emphasizes the potential of pervasive sensing technology to improve patient outcomes by enabling real-time adaptation of pain medications, personalizing analgesia choice, and improving assessments of delirium and mobility.

As another example of home-based health monitoring, Laidig et al. proposed a set of algorithms that use two foot-worn IMUs to accurately determine spatiotemporal gait parameters essential for clinical gait assessment without requiring magnetometers or precise sensor mounting. The proposed methods offer a calibration-free and unsupervised approach to gait assessment in daily-life environments. The algorithms are validated on a broad dataset of healthy subjects and orthopedic and neurological patients walking on a treadmill, showing a strong correlation and high accuracy for walking speeds and pathologies.

Folkvord et al. described a research study investigating the effectiveness of self-tracking apps on psychological outcomes related to self-empowerment and better-informed lifestyle decision-making. The study will consist of three parts: a systematic review of experimental evidence, a longitudinal field experiment comparing exercise programs with and without the aid of the self-tracking app Strava, and interviews with a subset of participants to gather qualitative data. The authors hope this study will provide a better understanding of the effects of self-tracking apps on psychological outcomes and inform the development of more effective health and activity monitoring tools.

A study by Kaye et al. examined changes in Chronic obstructive pulmonary disease (COPD) patients' COPD Assessment Test (CAT) scores and short-acting beta-agonists (SABA) inhaler use over six months in a digital health program that provided electronic medication monitors (EMMs) and a smartphone app. The results showed that CAT scores improved by a mean of −0.9 points, and SABA use decreased by −0.6 puffs per day, with more remarkable improvement observed in patients with a higher disease burden. These findings suggest that passively collected data from EMMs can be used to monitor disease burden and treatment outcomes in COPD patients.

In another application of mobile technology to pulmonary medicine, an article by Dave and Gupta discussed machine learning (ML) in contact tracing apps during the COVID-19 pandemic. ML can use data from these apps to forecast virus spread and identify vulnerable groups. Still, it's essential to ensure the accuracy, reliability, and lack of biases in the dataset to make reliable predictions. The article presents two requirements to meet international data quality standards for ML. It identifies where these requirements can be met, given varying contact tracing apps and smartphone usage in different countries. Lastly, this approach's advantages, limitations, and ethical considerations are discussed.

As we see the use of wearable and digital technologies being applied to the treatment and monitoring of mental health disorders—particularly for outpatient care—we are starting to see more research exploring how wearable data can be used to inform mental health treatment. A study by Abbas et al. aimed to examine whether machine learning-based visual and auditory digital markers can quantify response to antidepressant treatment (ADT) with selective serotonin reuptake inhibitors (SSRIs) and serotonin–norepinephrine uptake inhibitors (SNRIs). The researchers used automated smartphone tasks to measure facial, vocal, and head movement characteristics across four weeks of treatment for MDD patients. Results show that digital markers associated with MDD demonstrate validity as measures of treatment response, with significant changes in the MADRS and multiple digital markers, including facial expressivity, head movement, and amount of speech.

In conclusion, the recent advancements in mobile and wearable devices have opened tremendous opportunities to improve practical health applications and enable more accurate clinical decision-making. These technologies have greatly enhanced clinical capabilities, from gait assessment to blood pressure monitoring and COVID signs detection. Applying machine learning and statistical models to different health applications has shown promising results. The progress in mobile and wearable devices highlights the central role that model development will play in shaping the future of healthcare. This multidisciplinary research topic covers a range of areas, including machine learning, signal quality, patient reporting, mobile contact tracing, virtual assessment, and computational approaches for health monitoring.

